# Identifying gene regulatory modules of heat shock response in yeast

**DOI:** 10.1186/1471-2164-9-439

**Published:** 2008-09-23

**Authors:** Wei-Sheng Wu, Wen-Hsiung Li

**Affiliations:** 1 Department of Evolution and Ecology, University of Chicago, 1101 East 57th Street, Chicago, IL, 60637, USA; 2 Research Center for Biodiversity and Genomics Research Center, Academia Sinica, Taipei, Taiwan

## Abstract

**Background:**

A gene regulatory module (GRM) is a set of genes that is regulated by the same set of transcription factors (TFs). By organizing the genome into GRMs, a living cell can coordinate the activities of many genes in response to various internal and external stimuli. Therefore, identifying GRMs is helpful for understanding gene regulation.

**Results:**

Integrating transcription factor binding site (TFBS), mutant, ChIP-chip, and heat shock time series gene expression data, we develop a method, called Heat-Inducible Module Identification Algorithm (HIMIA), for reconstructing GRMs of yeast heat shock response. Unlike previous module inference tools which are static statistics-based methods, HIMIA is a dynamic system model-based method that utilizes the dynamic nature of time series gene expression data. HIMIA identifies 29 GRMs, which in total contain 182 heat-inducible genes regulated by 12 heat-responsive TFs. Using various types of published data, we validate the biological relevance of the identified GRMs. Our analysis suggests that different combinations of a fairly small number of heat-responsive TFs regulate a large number of genes involved in heat shock response and that there may exist crosstalk between heat shock response and other cellular processes. Using HIMIA, we identify 68 uncharacterized genes that may be involved in heat shock response and we also identify their plausible heat-responsive regulators. Furthermore, HIMIA is capable of assigning the regulatory roles of the TFs that regulate GRMs and Cst6, Hsf1, Msn2, Msn4, and Yap1 are found to be activators of several GRMs. In addition, HIMIA refines two clusters of genes involved in heat shock response and provides a better understanding of how the complex expression program of heat shock response is regulated. Finally, we show that HIMIA outperforms four current module inference tools (GRAM, MOFA, ReMoDisvovery, and SAMBA), and we conduct two randomization tests to show that the output of HIMIA is statistically meaningful.

**Conclusion:**

HIMIA is effective for reconstructing GRMs of yeast heat shock response. Indeed, many of the reconstructed GRMs are in agreement with previous studies. Further, HIMIA predicts several interesting new modules and novel TF combinations. Our study shows that integrating multiple types of data is a powerful approach to studying complex biological systems.

## Background

Single-cell organisms such as yeasts constantly face changing or even harsh environments such as high temperature that threaten their survival or, at least, prevent them from performing optimally [1]. By organizing the genome into gene regulatory modules (GRMs), a yeast cell can coordinate the activities of many genes and carry out complex functions in response to high temperature. Therefore, identifying GRMs of heat response is instrumental for understanding cellular responses to heat shock.

The advances of high-throughput tools such as DNA microarray [2,3] and chromatin immunoprecipitation-DNA chip (ChIP-chip) [4,5] have made the computational reconstruction of GRMs of a yeast cell possible. Several module inference methods have been proposed. Lee *et al*. [6] performed ChIP-chip experiments on 106 TFs in yeast and discovered six types of network motifs in yeast gene regulation. Using microarray data, Segal *et al*. [7] developed a probabilistic model to identify yeast GRMs. Later, three studies were conducted to combine ChIP-chip and gene expression data to identify yeast GRMs. First, Xu *et al*. [8] extended Segal *et al*.'s probabilistic model to incorporate ChIP-chip data. Second, Bar-Joseph *et al*. [9] developed GRAM to identify rich medium gene regulatory modules. Third, Wu *et al*. [10] developed MOFA to identify GRMs of yeast cell cycle. More data sources were used in more recent studies. Kato *et al*. [11] identified GRMs of yeast cell cycle by combining sequence, ChIP-chip and gene expression data. Lemmens *et al*. [12] developed ReMoDiscovery to identify GRMs of the yeast cell cycle and yeast stress response by combining motif information, ChIP-chip and gene expression data. Tanay *et al*. [13] applied a graph theoretic approach and developed SAMBA to reveal the modular organization of the yeast regulatory system by combining protein interactions, growth phenotype data, ChIP-chip, and gene expression data. However, all these module inference algorithms are statistics-based methods, treating dynamic time series gene expression data the same way as static steady state gene expression data. That is, they do not consider the dependency between different time points of a time series and thus do not utilize the dynamic nature of time series data. Because many time series gene expression data sets are now available in the public domain [14-18], it is desirable to develop a module inference method that can utilize the dynamic nature of time series gene expression data. The aim of this study is to develop a module inference method that suits this need.

**Figure 1:**
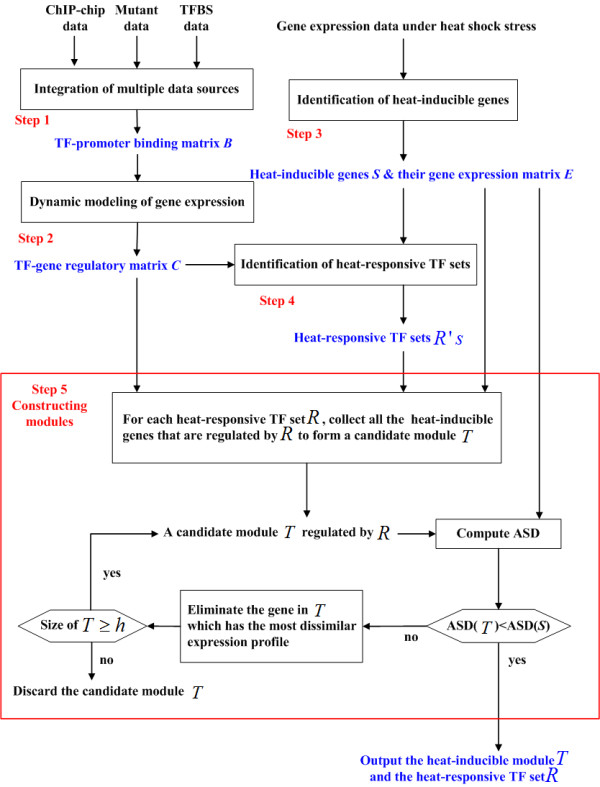
The flowchart of HIMIA.

By combining current transcription factor binding site (TFBS) [19,20], mutant [20], ChIP-chip [21], and heat shock time series gene expression data [18], we developed a module inference method, called Heat-Inducible Module Identification Algorithm (HIMIA), to reconstruct heat-inducible GRMs in yeast (see Figure 1). HIMIA is divided into five steps. First, three independent data sources (ChIP-chip, mutant, and TFBS data) are used to construct a high-confidence TF-promoter binding matrix (see Methods for details). From the TF-promoter binding matrix, all the TFs that bind to any specific target gene can be inferred. Second, using heat shock time series gene expression data, a dynamic system model of gene regulation is applied to describe how a target gene's expression under heat shock is controlled by the TFs that bind to its promoter (see Appendix for details). A dynamic system model is capable of utilizing the dynamic nature of time series gene expression data, making it different from the static statistics-based models in previous studies [7-13]. After the dynamic system modeling, the TFs that have significant regulatory effects on the target gene's expression can be extracted from all TFs that bind to the target gene. From this procedure, a high-confidence TF-gene regulatory matrix is constructed. Each TF-gene regulatory relationship in this matrix is supported by at least three independent data sources (gene expression and TFBS data plus ChIP-chip or/and mutant data). Third, candidate heat-inducible genes are identified. A gene is said to be heat-inducible if at least two time points of its gene expression profile measured under heat shock are induced by at least three folds compared to that under the unstressed condition. Causton *et al*. [18] defined a candidate heat-inducible gene by the criterion that at least one time point of its gene expression profile shows a change fold of at least three. In order to reduce the false positives, we use a criterion that requires at least two time points with a change fold of at least three. Fourth, using the list of candidate heat-inducible genes and the high-confidence TF-gene regulatory matrix, heat-responsive TF sets can be inferred by statistical methods. A TF set is said to be heat-responsive only if a significant portion of the targets that are co-regulated by all the TFs in the TF set is heat-inducible. The hypergeometric distribution is used to test the statistical significance. Fifth, for each heat-responsive TF set, we collect all their regulatory targets that are heat-inducible to form a candidate heat-inducible GRM. That is, a heat-inducible GRM consists of a set of heat-inducible genes that are regulated by the same set of heat-responsive TFs. Because genes in the same GRM are regulated by the same set of TFs, their gene expression profiles should be more similar to each other than those of a set of genes that are not in a single GRM. Therefore, for each candidate GRM we further extract a subset of genes whose gene expression profiles are more coherent than that of the set of all heat-inducible genes which are regulated by different sets of TFs. Finally, this subset of highly coherent genes forms a heat-inducible GRM that is regulated by the same set of heat-responsive TFs.

## Results

**Figure 2:**
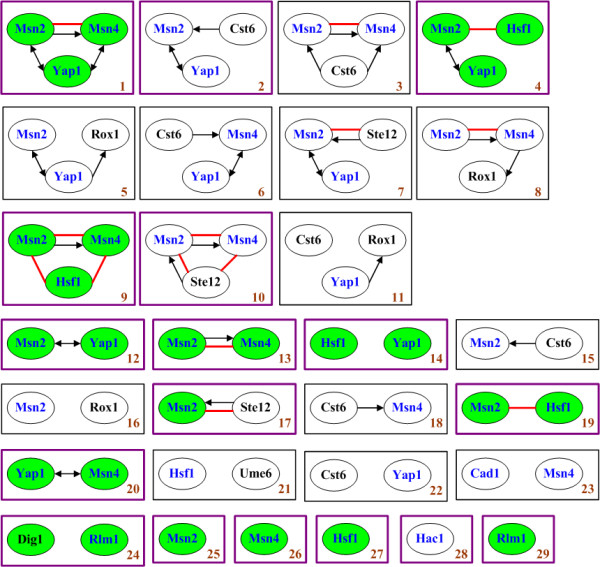
**The 29 GRMs identified in this study**. Each rectangle stands for a module and the ovals in a rectangle indicate the TFs that regulate the module. A TF name is colored blue if it is known to be involved in heat shock response but black otherwise. The periphery of a rectangle is colored purple if the module has at least one enriched MIPS functional category but black otherwise. An oval is colored green if the TF's function is consistent with at least one of the module's enriched MIPS functional categories. Two ovals are connected by an undirected red line if these two TFs have physical interactions indicated by the current protein-protein interaction data [22]. Two ovals are connected by a directed black line if the two TFs have genetic interactions indicated by mutant or ChIP-chip data [20,21]. For example, Msn2→Yap1 means that either TF Msn2 binds to the promoter of gene *YAP1 *or the disruption of TF Msn2 results in a significant change of the expression of gene *YAP1*.

By integrating TFBS, mutant, ChIP-chip, and gene expression data, HIMIA identified 29 GRMs, which in total contain 182 heat-inducible genes regulated by 12 heat-responsive TFs (see Figure 2 and Additional file 1). According to the literature [19-23,25-33,35], 108 of the 182 genes and 7 of the 12 TFs are known to be involved in heat shock response.

### Validation of the identified modules

Several lines of evidence support that HIMIA identifies biologically relevant heat-inducible GRMs. First, by the virtue of our method, each TF-gene regulatory relationship in a module is of high confidence because it is supported by at least three independent data sources (gene expression and TFBS data plus ChIP-chip or/and mutant data). Second, each module is assigned to at least one TF that is known to be involved in heat shock response (see Figure 2). Third, in the 24 modules that are controlled by more than one TF, 70% (32/46 counting multiplicity) of the TFs in the same module have physical or genetic interactions [20-22] (see Figure 2). Fourth, on average, 67% of genes in a heat-inducible GRM are known to be involved in heat shock response (see Additional file 1). Fifth, 59% (17/29) of the identified modules include groups of genes that function in the same cellular process: each of these modules contains at least one over-represented MIPS functional category [23] with the adjusted *p*-value < 0.05 (after the Bonferroni correction for multiple tests [24]) using the cumulative hypergeometric distribution (see Figure 2 and Additional file 1). Finally, the modules are generally accurate in assigning TFs to sets of genes whose functions are consistent with the TFs' known roles. For the 17 modules that contain groups of genes enriched in the same cellular process, we found that the regulatory functions of the 76% (26/34 counting multiplicity) of the TFs are consistent with one of their modules' over-represented functional categories (see Figure 2). Taken together, these results provide evidence that HIMIA identifies not only sets of biologically related heat-inducible genes, but also heat-responsive TFs that individually or collectively regulate these genes.

### Identification of important heat-responsive TFs

We identified 12 heat-responsive TFs, 7 (Msn2, Msn4, Hsf1, Yap1, Hac1, Rlm1 and Cad1) of which are known to be involved in heat shock response (see Table 1). Our findings are supported by the literature. First, Msn2 and Msn4 are known to regulate the general stress response in yeast. They regulate the expression of many genes in response to several stresses, including heat shock, osmotic shock, oxidative stress, low pH, glucose starvation, sorbic acid and high ethanol concentrations, by binding to the stress response element (STRE) located in the promoters of these genes [25-27]. Second, Hsf1 is the well-known heat shock factor that binds to the heat shock element (HSE) to regulate the transcription of many heat-inducible genes, including genes involved in protein folding, detoxification, energy generation, carbohydrate metabolism, and cell wall organization [25,27,28]. All these gene products are important for cell to counteract the deleterious effects of heat. Third, Yap1, a well-known oxidative shock factor, is also known to be involved in heat shock response [29]. For example, Yap1 induces the expression of *GSH1 *and *GSH2 *to synthesize glutathione in heat shock response [30]. Fourth, heat stress can cause unfolded proteins to accumulate in the endoplasmic reticulum (ER), triggering the unfolded protein response (UPR). Hac1 binds to the UPR element (UPRE) to regulate genes that are involved in UPR [31]. Fifth, heat stress causes a weakening of cell wall and membrane stretching which stimulates the protein kinase C (PKC) pathway [25]. Rlm1, a component of the PKC pathway, is then activated to perform the function of maintaining the cell wall integrity [32]. Finally, Cad1, an AP-1 like bZIP transcriptional activator involved in stress responses (e.g. heat shock response, pleiotropic drug resistance), controls a set of genes involved in stabilizing proteins [33]. The involvement of Cad1 in stress responses was also identified by Segal *et al*. [7].

**Table 1:** The number of inferred heat-inducible target genes regulated by each of the 12 heat-responsive TFs

TF name	# of inferred heat-inducible target genes
**Msn2**	73
**Msn4**	67
**Hsf1**	64
**Yap1**	48
**Hac1**	29
**Rlm1**	23
Cst6	14
Ste12	14
Rox1	13
Ume6	8
Dig1	7
**Cad1**	6

Seven well-known heat-responsive TFs are bold-faced and colored blue. The TFs are ordered by the number of their inferred heat-inducible target genes.

**Figure 3:**
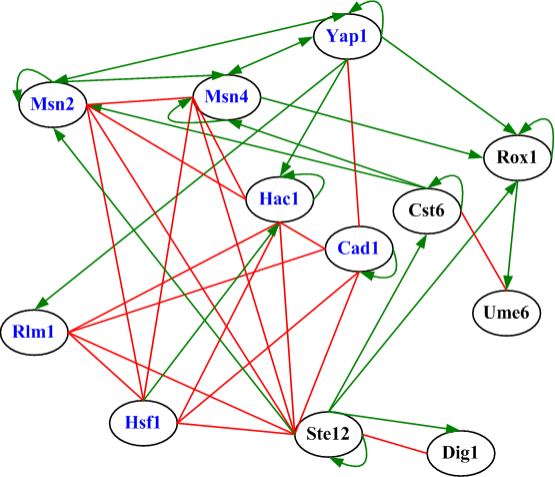
**The interaction network formed by the 12 identified heat-responsive TFs**. An oval indicates an identified heat-responsive TF. A TF name is colored blue if it is known to be involved in heat shock response but black otherwise. Two ovals are connected by an undirected red line if the two TFs have physical interactions indicated by the current protein-protein interaction data [22]. Two ovals are connected by a directed green line if these two TFs have genetic interactions indicated by the mutant or ChIP-chip data [20,21]. Note that the 12 identified heat-responsive TFs form a highly connected network of interactions.

In addition to the above seven known heat-responsive TFs, five novel heat-responsive TFs (Rox1, Cst6, Ume6, Ste12 and Dig1) have also been identified by HIMIA. Rox1 contains a high-mobility group (HMG) domain that is responsible for DNA bending activity [29]. Cst6 and Ume6 regulate genes involved in the cell cycle and DNA processing [23]. Ste12 and Dig1 are involved in the regulation of mating-specific genes and the invasive growth pathway [29]. Identification of these five TFs as heat-responsive TFs suggests that heat shock response may have crosstalk with other cellular processes. It is known that the cell cycle transiently arrests during a heat shock stress [25], validating our proposal that heat shock response may have crosstalk with the cell cycle process. Moreover, these five novel heat-responsive TFs and the other seven known heat-responsive TFs form a highly connected network of interactions, supporting our prediction that these five novel heat-responsive TFs may play a role in heat shock response (see Figure 3). This dense network of interactions also suggests that different combinations of a fairly small number of heat-responsive TFs may be sufficient to regulate a large number of genes involved in heat shock response.

Taken together, these results suggest that HIMIA is effective for identifying TFs that play a role in heat shock response or are involved in other cellular processes that may crosstalk with heat shock response.

### Identification of important genes involved in heat shock response

Disruption of a large number of cellular assemblies and processes, an increased protein unfolding and aggregation, and membrane structure alterations are paramount in cells exposed to high temperature. Heat shock response serves to counteract these deleterious effects. Through it cells increase their thermotolerance or ability to withstand heat stress [25].

Many events occur in yeast cells during heat shock response. First, heat shock proteins (HSPs) are rapidly synthesized. Many HSPs function as protein chaperones, so named because of their ability to bind to partially unfolded proteins to protect them from degradation or aggregation [1]. HIMIA successfully identified heat shock protein genes *HSP10 *and *HSP60 *(both in module 27) to be regulated by heat-shock transcription factor Hsf1 and *HSP12 *and *HSP78 *(both in module 25) to be regulated by the general stress factor Msn2. In addition, *HSP31*, *HSP104*, *SSA1*, *SSA4 *and *SSE2 *(all in module 19) are identified to be regulated by both Hsf1 and Msn2. Other genes involved in the protein folding or refolding are also found. For example, *EUG1*, *LHS1*, *SCJ1 *and *ERO1 *(all in module 28) are regulated by Hac1, a TF known to be involved in protein unfolding response [31]. Second, heat shock causes the extremely rapid accumulation of a large cytoplasmic pool of trehalose. Trehalose is one of the most effective substances known for preservation of membranous structures and enzyme activities during heating. One of the major roles for Msn2/4 in heat shock response is to regulate the expression of genes required for the synthesis of trehalose [25]. HIMIA successfully identified three genes for trehalose synthease subunits: *TSL1 *(in module 25), *TSP1*, and *NTH1 *(both in module 26) are indeed regulated by Msn2 or Msn4. Third, heat shock increases the synthesis of certain components of the ubiquitination system for intracellular protein turnover, indicating a much greater requirement for turnover of abnormal proteins in cells recovering from heat stress [25]. HIMIA successfully identified several genes that are known to be involved in protein degradation. *DER1 *and *PBI2 *(both in module 28) are identified to be regulated by Hac1. *APG12*, *UBC8*, *JEM1 *and *UBI4 *(all in module 27) are regulated by Hsf1. *LAP4 *and *ASI1 *(both in module 25) are regulated by Msn2. *ATG8 *and *YSP3 *(both in module 29) are regulated by Rlm1. Fourth, high temperature causes the weakness of cell walls and induces the expression of genes that are involved in cell wall biogenesis and maintenance [25]. HIMIA successfully identified seven genes (*CWP1*, *GFA1*, *KTR2*, *FLC2*, *CHS3*, *HKR1 *and *SLT2*) known to be involved in the cell wall biogenesis and maintenance [29]. All these seven genes are in module 29 and regulated by Rlm1, an important cell integrity maintenance factor [32]. Finally, it is known that during heat shock cells induce a variety of genes related to carbohydrate metabolism, fatty acid metabolism, respiration and others [25]. HIMIA successfully identified genes that are involved in these cellular processes. For example, *HXK1*, *GLK1 *and *GPH1 *(all in module 25) involved in glucose metabolism are identified to be regulated by Msn2. *FAA1 *and *FAA4 *(both in module 27) involved in fatty acid metabolism are identified to be regulated by Hsf1. *CYC7 *and *COX20 *(both in module 26) involved in respiration are identified to be regulated by Msn4. The known functions of these genes hint the cellular processes that may be affected in response to heat shock, and suggest mechanisms the cell uses to protect itself in the face of a heat stress [25].

### Annotating uncharacterized genes

Among the 182 identified heat-inducible genes, 68 genes have unknown function according to the *Saccharomyces *Genome Database [29]. We suggest most if not all of these genes are involved in heat shock response. Our predictions are supported by the fact that all these 68 genes are induced by more than three folds at least at two time points of their expression profiles under heat shock. Moreover, all these 68 genes are regulated by known heat-responsive TFs and the TF-gene regulatory relationships are supported by at least three independent data sources (gene expression and TFBS plus ChIP-chip or/and mutant data). As shown in Additional file 1, 14 of these genes are regulated by Hac1, 25 by Hsf1, 24 by Msn2, 20 by Msn4, 17 by Yap1, and so on.

As an example, it is known that the weakening of cell wall during high temperature stimulates the cell integrity pathway [25]. *PRM5 *is known to be induced in the cell integrity pathway but its molecular function is unknown [29]. HIMIA identified *PRM5 *(in module 29) to be regulated by Rlm1, an important cell wall integrity maintenance factor, suggesting that *PRM5 *is indeed involved in the cell wall maintenance during heat shock. Moreover, we successfully identified the putative heat shock protein gene *YRO2 *(in module 26) to be regulated by Msn4. In addition, putative genes that may be involved in protein degradation are also found. For example, *HBT1 *and *RIM20 *(both in module 26) are identified to be regulated by Msn4. *MGR1 *and *ADD37 *(both in module 28) are identified to be regulated by Hac1. All these examples show that these uncharacterized genes may play a role in heat shock response. However, further experimental validations are needed to confidently annotate these uncharacterized genes as heat-inducible genes.

### Assigning regulatory roles of TFs that regulate a GRM

We can assign the regulatory roles of TFs that regulate a module. A TF is said to be an activator of a module if the *p*-value for observing so many TF-gene pairs in the module each with a significant positive correlation (within the 5% right tail of the distribution of correlation) is less than 0.001. The *p*-value is the probability that an observation would be made by chance, and is calculated using the cumulative binomial distribution [10]: $P(x\geq n_{0})=\sum_{x=n_{0}}^{N}\left[\frac{N!}{x!(N-x)!}\right]p_{0}^{x}{\left(1-p_{0}\right)}^{N-x}$

where *N *is the total number of genes in a module, *n*_0 _is the number of genes in the module that show a significant positive correlation in expression with the TF, and *p*_0 _is the probability of observing an arbitrary gene in the genome that has a significant positive correlation in expression with the TF. As shown in Table 2, we assigned Cst6 as an activator of four modules, Hsf1 an activator of three modules, Msn2 an activator of five modules, Msn4 an activator of two modules, Rlm1 an activator of two modules and Yap1 an activator of four modules.

**Table 2:** Identified activators of GRMs

Activator identified	Module # (*p*-value)
Cst6	#2 (3.7 × 10^-4^), #3 (3.7 × 10^-4^), #15 (6.4 × 10^-5^), #18 (1 × 10^-3^)
Hsf1	#14 (6.8 × 10^-5^), #21 (3.7 × 10^-4^), #27 (2.4 × 10^-13^)
Msn2	#12 (8.5 × 10^-5^), #13 (4.1 × 10^-5^), #17 (3.3 × 10^-5^), #19 (8.6 × 10^-4^), #25 (5.8 × 10^-13^)
Msn4	#13 (1.2 × 10^-8^), #26 (<1 × 10^-13^)
Rlm1	#24 (1.9 × 10^-4^), #29 (2 × 10^-10^)
Yap1	#1 (9.7 × 10^-6^), #12 (9 × 10^-9^), #14 (5.3 × 10^-7^), #20 (3.3 × 10^-7^)

A TF is said to be an activator of a module if the *p*-value for observing so many TF-gene pairs in the module each with a significant positive correlation is less than 0.001.

### Refining the clusters of the genes involved in the same cellular process

**Figure 4:**
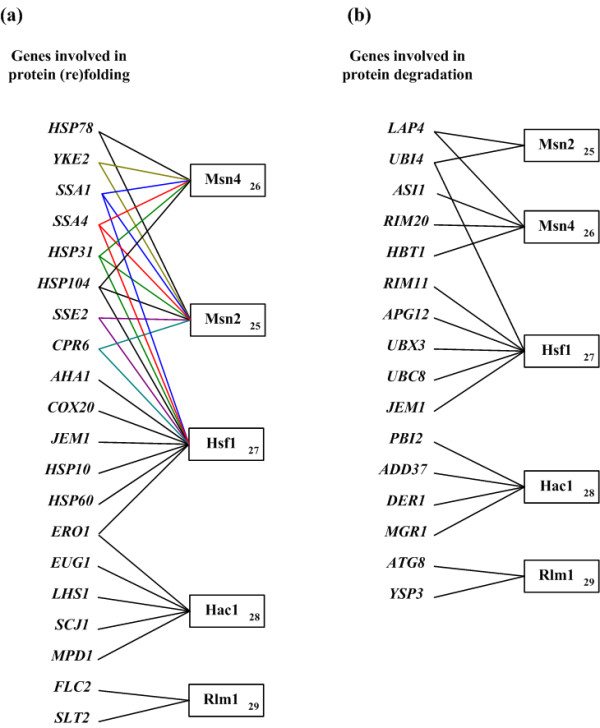
**Refining clusters of genes involved in the same cellular process**. (A) Refining the cluster of genes involved in protein (re)folding. HIMIA assigns the 20 identified genes that are involved in protein (re)folding into five modules. (B) Refining the cluster of genes involved in protein degradation. HIMIA assigns the 16 identified genes that are involved in protein degradation into five modules.

Heat shock causes an increased protein unfolding and aggregation in a cell. Thus, many genes that are involved in protein (re)folding are induced to bind to partially unfolded proteins to protect them from degradation or aggregation [25]. Among the 182 identified heat-inducible genes, 20 genes are known to be involved in protein (re)folding. Although these genes are functionally similar, they may be under different transcriptional controls. Indeed, HIMIA assigns these 20 genes into five modules (see Figure 4a). For example, *HSP10 *and *HSP60 *are identified to be regulated by Hsf1, *HSP78 *by Msn2/4, *HSP31 *and *HSP104 *by both Hsf1 and Msn2/4, and so on.

Denatured proteins that cannot be properly refolded are targeted for degradation by ubiquitination, so it is not surprising that genes involved in protein degradation are induced under heat shock [25]. Among the 182 identified heat-inducible genes, 16 genes are known to be involved in protein degradation. Although these genes are functionally similar, they may be under different transcriptional controls. HIMIA assigns these 16 genes into five modules (see Figure 4b). For example, *PBI2*, *ADD37*,*DER1 *and *MGR1 *are identified to be regulated by Hac1, *ATG8 *and *YSP3 *by Rlm1, and so on.

Therefore, HIMIA can refine the cluster of genes involved in protein (re)folding and degradation and can provide a better understanding of how the cell regulates the complex expression program of these genes.

## Discussion

### Performance comparison with existing methods

Several module inference methods have been proposed. GRAM [9] and MOFA [10] are intuitive algorithms to identify yeast GRMs by combining ChIP-chip and gene expression data. ReMoDiscovery [12] is an intuitive algorithm to identify yeast GRMs by combining motif information, ChIP-chip, and gene expression data. SAMBA [13] uses a graph theoretic approach to identify GRMs by combining four data sources (ChIP-chip, gene expression, protein interactions, and growth phenotype data). All these four module inference algorithms are statistics-based methods. On the contrary, HIMIA is a dynamic system model-based method that integrates four data sources (ChIP-chip, time series gene expression, mutant, and TFBS data). Since HIMIA is different from the four pervious methods, a performance comparison was conducted. We tested the ability of each of these five algorithms to retrieve the known stress-responsive TFs annotated in the MIPS database [23]. Performance comparison was based on the Jaccard similarity score [12,34], which scores the overlap between an algorithm's output and the list of known stress-responsive TFs (i.e., the true answers). Therefore, the higher the Jaccard similarity score, the better the ability of an algorithm to retrieve the known stress-responsive TFs. Table 3 shows that HIMIA has the highest Jaccard similarity score among the five tested algorithms.

**Table 3:** Performance comparison of five module inference tools

Algorithm name	TP	FP	FN	Jaccard similarity score
HIMIA	7	5	25	0.19
ReMoDiscoery	11	38	21	0.16
GRAM	13	56	19	0.15
MOFA	8	32	24	0.13
SAMBA	10	54	22	0.12

Performance comparison was based on the Jaccard similarity score [12,34], which scores the overlap between an algorithm's output and the list of known stress-responsive TFs. Specifically, the score is defined as TP/(TP+FP+FN) [12,34], where TP stands for true positives, FP for false positives, and FN for false negatives. Note that the higher the Jaccard similarity score, the better the ability of an algorithm to retrieve the known stress-responsive TFs.

### Randomization test and comparison with a null model

Two randomization tests were performed to show that the output of HIMIA is statistically meaningful. First, we randomly permuted the gene expression data 1000 times. Second, we randomly permuted the ChIP-chip data 1000 times. When either of the two randomized data sets was used as the input, HIMIA could no longer effectively identify the known stress-responsive TFs (see Table 4). This means that the output of HIMIA is statistically meaningful and is far beyond random expectation.

**Table 4:** Randomization tests of HIMIA

	Jaccard similarity score
HIMIA (using original input data)	0.19
HIMIA (using randomized gene expression data)	0.025 (mean), 0.023 (standard deviation)
HIMIA (using randomized ChIP-chip data)	0.009 (mean), 0.016 (standard deviation)

Two randomization tests were performed. First, we randomly permuted the gene expression data 1000 times. Second, we randomly permuted the ChIP-chip data 1000 times. When either of the two randomized data sets was used as the input, HIMIA could no longer effectively identify the known stress-responsive TFs, indicating that the output of HIMIA is statistically meaningful and is far beyond random expectation.

In addition, we compared the performance of HIMIA and the null model to retrieve the known stress-responsive TFs annotated in the MIPS database [23]. The null model was defined as using the same input data (mutant, ChIP-chip, TFBS, and gene expression data) but only the overlap was calculated instead of going through the iterative procedure described on Figure 1. The simulation result shows that HIMIA has a better ability in retrieving the known stress-responsive TFs than does the null model (Jaccard similarity scores 0.19 v.s. 0.17).

### Parameter settings of HIMIA

The choice of the relaxing the *p*-value to 0.01 (in Step 1 of HIMIA) has a biological meaning. Two previous papers [6,21] used a statistical error model to assign a *p*-value of the binding relationship of a TF-promoter pair. They found that if *p *≤ 0.001, the binding relationship of a TF-promoter pair is of high confidence and can usually be confirmed by promoter-specific PCR. If *p *> 0.01, the binding relationship of a TF-promoter pair is of low confidence and cannot be confirmed by promoter-specific PCR most of the time. However, if 0.001 <*p *≤ 0.01, the binding relationship of a TF-promoter pair is ambiguous and can be confirmed by promoter-specific PCR in some cases but not in the other cases. One of our aims in this study was to solve this ambiguity, so we chose 0.01 to be the relaxed *p*-value. However, we added the requirement that the promoter must contain one or more binding sites of the TF.

In the original paper, Causton *et al*. [18] defined a candidate heat-inducible gene by the criterion that at least one time point of its gene expression profile showed a change fold of at least three. In order to reduce the chance of including false positives, we used a criterion that at least two time points showed a change fold of at least three. Using this criterion, 528 genes were identified as candidate heat-inducible genes. Of course, some false positives might still exist among these 528 genes. In the subsequent steps, however, HIMIA further refined this set of candidate heat-inducible genes using four independent data sources (mutant, ChIP-chip, TFBS, and time series gene expression data). Finally, only 182 genes were identified heat-inducible genes, which were classified into 29 heat-inducible GRMs regulated by heat-responsive TFs. Among the 182 identified genes, 108 are known to be involved in heat shock response. We provided several lines of evidence to support the biological relevance of the 29 identified heat-inducible GRMs (see Results section for details). Therefore, the possible existence of false positives should not be a serious problem in HIMIA.

### Genes in the heat-inducible GRMs

HIMIA identified 29 heat-inducible GRMs. On average, 67% of the genes in a heat-inducible GRM are known to be involved in heat shock response, 17% of the genes in a GRM are uncharacterized genes (i.e., proteins with unknown functions), and 16% of the genes in a GRM are annotated in other cellular processes. Although we cannot claim that all genes in the last two categories are heat-inducible genes, our predictions are supported by three observations. First, all these genes are induced by more than three folds at least at two time points of their expression profiles under heat shock. Second, all these genes are regulated by at least one known heat-responsive TFs in the corresponding GRMs, and the TF-gene regulatory relationships are supported by at least three independent data sources (gene expression and TFBS plus ChIP-chip or/and mutant data). Third, it is known that during heat shock yeast cells express a variety of genes related to carbohydrate metabolism, fatty acid metabolism, energy generation, respiration, signaling pathways (e.g. PKC and cell integrity pathways) and others [1,25]. For the known genes in a GRM that have not been annotated in heat shock response, most of them are related to one of the cellular processes mentioned above. This provides indirect evidence for these genes to be possibly triggered by heat shock response. However, further experimental validations are needed to reliably claim that these predicted heat-inducible genes do play a role in heat shock response.

We also found that genes with diverse functions may be in the same GRM. As an example, Hsf1, Msn2, and Yap1 are known heat-responsive TFs [1,23,25-30,35] and eight genes (*HSP31*, *HSP104*, *SSA4*, *SSE2*, *CPR6*, *SPI1*, *TKL2*, and *YHR087W*) are in the module {Hsf1, Msn2, Yap1} (module 4). *HSP31*, *HSP104*, *SSA4 *and *SSE2 *are genes encoding heat shock proteins that function as protein chaperones [29]. *CPR6 *encodes a protein that binds to Hsp82 and contributes to chaperone activity [29]. *SPI1 *encodes a protein that is involved in cell wall biogenesis [35]. *TKL2 *is involved in carbon metabolism and is induced in response to heat shock [27]. *YHR087W *is an uncharacterized gene with unknown function [29]. It is not surprising that we find genes encoding HSPs, cell wall biogenesis protein, and carbon metabolic proteins in a GRM because they are all needed when yeast cells are subjected to heat shock [1,25]. HSPs function as protein chaperones, so named because of their ability to bind to partially unfolded proteins to protect them from degradation or aggregation. High temperature causes the weakness of cell wall and induces the expression of genes that are involved in cell wall biogenesis and maintenance. It is known that heat stress imposes large demands for energy (ATP) generation by the cell [25]. In short, heat shock response is multifaceted, involving the expression of genes with diverse functions.

## Conclusion

We developed a method, called Heat-Inducible Module Identification Algorithm (HIMIA), for reconstructing GRMs of heat shock response in yeast by integrating current ChIP-chip, mutant, TFBS, and time series gene expression data. Unlike previous module inference tools which are static statistics-based methods, HIMIA is a dynamic system model-based method that utilizes the dynamic nature of time series gene expression data. HIMIA identified 29 GRMs, which in total contain 182 heat-inducible genes regulated by 12 heat-responsive TFs. The literature indicates that 108 of the 182 genes and 7 of the 12 TFs are known to be involved in heat shock response. The biological relevance of each inferred GRM was validated by using the literature, enrichment for genes in the same MIPS functional category, protein-protein interaction data, and so on. Our analysis suggests that different combinations of a fairly small number of heat-responsive TFs may be responsible for regulating a large number of genes involved in heat shock response and that there may exist crosstalk between heat shock response and other cellular processes. In addition, HIMIA suggested that 68 uncharacterized genes may be involved in heat shock response and it also identified their plausible heat-responsive regulators. Furthermore, HIMIA is capable of assigning the regulatory roles of the TFs that regulate GRMs and Cst6, Hsf1, Msn2, Msn4, and Yap1 are found to be activators of several GRMs. In addition, HIMIA refined two clusters of genes that are involved in heat shock response and provided a better understanding of how the complex expression program of heat shock is regulated. Finally, we showed that HIMIA outperformed four current module inference tools (GRAM, MOFA, ReMoDisvovery, and SAMBA), and we conducted two randomization tests to show that the output of HIMIA is statistically meaningful.

## Methods

### Data sets and data preprocessing

We use four kinds of data in this study. First, the ChIP-chip data are from Harbison *et al*. [21]. They used genome-wide location analysis to determine the genomic occupancy of 203 TFs in rich media conditions and, for 84 of these TFs, in at least one of 12 other environmental conditions. Second, the TFBS data are from MacIsaac *et al*.'s study [19] and the YEASTRACT database [20]. MacIsaac *et al*. used evolutionarily conservative criteria to computationally identify the binding sites of many TFs. The YEASTRACT database includes a set of computational tools that can be used to identify complex motifs over-represented in the promoters of co-regulated genes. Third, the mutant data are from the YEASTRACT database [20]. The mutant data can tell us which gene's expression was changed significantly owing to the deletion (or mutation) of the gene that encodes a TF. The evidence may come from detailed gene by gene analysis or genome-wide expression analysis. Finally, the time series gene expression data under heat shock stress are from Causton *et al*.'s study [18]. Samples for all genes in the yeast genome are collected at six time points (0, 15, 30, 45, 60, 120 minute). That is, each gene has a 6-timepoint gene expression profile. The cubic spline method [36] is then used to interpolate extra data points into the original time profile. Note that genes that have missing values in their original time profiles are discarded in our study. We did not use Gasch *et al*.'s gene expression data under heat shock stress [17] because 41% (2509/6152) of the genes in the yeast genome had missing values in their time profiles.

### Average standard deviation (ASD)

To check the expression coherence of a set of genes, ASD is used [11]: *ASD *≜ *m*(*σ*(*e*_*g*,*i*_)), where *e*_*g*,*i *_is the normalized expression level of gene *g *at time *i*, *σ *is the standard deviation over genes, and *m *is the average over time. The lower the ASD is, the closer the expression levels are to the average, i.e., the more coherent in expression profiles of the genes in a set are.

### Heat-Inducible Module Identification Algorithm (HIMIA)

HIMIA are divided into five steps.

#### Step 1

Construction of a high-confidence TF-promoter binding matrix *B *= [*b*_*i*,*j*_]

In this matrix, *b*_*i*,*j *_= 1 if (1) the *p-*value for TF*j *to bind the promoter of gene *i *is ≤ 0.01 in the ChIP-chip data and the promoter of gene *i *contains one or more binding sites of TF*j *or (2) the disruption of TF*j *results in a significant change of the expression of gene *i *and the promoter of gene *i *contains one or more binding sites of TF*j*. Otherwise, *b*_*i*,*j *_= 0

#### Step 2

Construction of a high-confidence TF-gene regulatory matrix *C *= [*c*_*i*,*j*_]

In this matrix, *c*_*i*,*j *_= 1 if *b*_*i*,*j *_= 1 and if TF*j *is shown by the dynamic model to have a large regulatory effect on the expression of gene *i *(see Appendix for details). Otherwise, *c*_*i*,*j *_= 0

#### Step 3

Identification of heat-inducible genes and construction of their gene expression matrix *E *= [*e*_*p*,*q*_]

A gene is called a heat-inducible gene if at least two time points of its gene expression profile measured under heat shock are induced by at least three folds compared to that under the unstressed condition. We then collect all the time profiles of the identified heat-inducible genes to form a matrix *E *= [*e*_*p*,*q*_], where *e*_*p*,*q *_is the expression value of the *p-*th heat-inducible gene at time point *q*.

#### Step 4

Identification of heat-responsive TF sets

The number of TFs in a TF set could be one, two or more. A TF set is said to be heat-responsive if a statistically significant portion of the target genes that are co-regulated by all the TFs in the TF set is heat-inducible. The hypergeometric distribution is used to test the statistical significance. For example, let *R *={TF_*u*_, TF_*v*_, TF_w_} be a TF set, *G *= {gene *k *| *c*_*k*,*u *_= *c*_*k*,*v *_= *c*_*k*,*w *_= 1} be the set of genes that are regulated by all the TFs in *R*, *S *be the set of heat-inducible genes in the yeast genome identified in Step 3, *T *= *G *∩ *S *be the set of heat-inducible genes that are regulated by all the TFs in *R*, and *Y *be the set of all genes in the yeast genome. Then the *p*-value for rejecting the null hypothesis (H_0_: *R *is not a heat-responsive TF set) is calculated as [37]:

$$p=P(x\geq \left|T\right|)=\sum_{x\geq \left|T\right|}\frac{\left(\begin{array}{c} \left|S\right|\\ x \end{array}\right)\left(\begin{array}{c} \left|Y\right|-\left|S\right|\\ \left|G\right|-x \end{array}\right)}{\left(\begin{array}{c} \left|Y\right|\\ \left|G\right| \end{array}\right)},$$

where |*G*| means the number of genes in set *G*. This *p*-value is then adjusted by the Bonferroni correction to represent the true alpha level in the multiple hypothesis testing [24]. Finally, *R *is said to be a heat-responsive TF set if the adjusted *p*-value *p*_*adjusted *_≤ 0.01

#### Step 5

Construction of heat-inducible GRMs

For each heat-responsive TF set *R *identified in Step 4, *T *forms a heat-inducible candidate GRM. That is, a heat-inducible candidate GRM *T *consists of a set of heat-inducible genes that are regulated by a heat-responsive TF set *R*. Because genes in the same GRM are regulated by the same set of TFs, their gene expression profiles should be more similar to each other than those of a set of genes that are not in a single GRM. Therefore, we require that the gene expression profiles of the genes in the same GRM be more coherent than those of the set of all heat-inducible genes, which are regulated by different sets of TFs. For measuring the expression coherence of a set of genes, ASD is used. The lower the ASD is, the more coherent the expression profiles of the genes in a set are. Therefore, if *ASD*(*T*) ≥ *ASD*(*S*) HIMIA iteratively eliminates genes in *T *starting from the one with the most dissimilar expression profile until *ASD*($\tilde{T}$) <*ASD*(*S*), where $\tilde{T}$ is the set of the remaining genes and *S *is the set of all heat-inducible genes identified in Step 3. That is, HIMIA tries to identify a subset of co-regulated heat-inducible genes whose gene expression profiles are more coherent than those of the set of all heat-inducible genes which are regulated by different sets of TFs. Finally, HIMIA outputs a GRM *M*(*R*) ≜ $\tilde{T}$ if $\tilde{T}$ contains more than a certain number of genes, say five. The above procedure goes over all heat-responsive TF sets *R*'s identified in Step 4.

### Jaccard similarity score

The Jaccard similarity score [12,34] was used to score the overlap between an algorithm's output and the list of known stress-responsive TFs. Specifically, it is defined as TP/(TP+FP+FN), where TP stands for true positives, FP for false positives, and FN for false negatives. Clearly, the higher the Jaccard similarity score, the better the ability of an algorithm to retrieve the known stress-responsive TFs.

## Appendix

### Dynamic system model of gene regulation

We consider the transcriptional regulatory mechanism of a target gene as a system with the regulatory profiles of several TFs as the inputs and the gene expression profile of the target gene as the output. The transcriptional regulation of a target gene is described by the following stochastic dynamic equation [38-40]:

(1) $$y[t+1]=\left(\sum_{i=1}^{N}d_{i}\cdot x_{i}[t]+k\right)-\lambda \cdot y[t]+\varepsilon [t]$$

where *y*[*t*] represents the expression profile of the target gene at time point *t*, *N *denotes the number of TFs that bind to the promoter of the target gene inferred from the TF-promoter binding matrix *B*, *d*_*i *_indicates the regulatory ability of TF *i*, *x*_*i*_[*t*] represents the regulatory profile of TF*i *at time point *t*, *k *represents the basal level induced by RNA polymerase II, *λ *indicates the degrading effect of the target gene's expression at present time point *y*[*t*] on the target gene's expression at next time point *y*[*t *+ 1] and *ε*[*t*] denotes the stochastic noise due to the modeling error and the measuring error of the target gene's expression profile. *ε*[*t*] is assumed to be a Gaussian noise with mean zero and unknown standard deviation *σ*. The biological meaning of Equation (1) is that *y*[*t *+ 1] (the target gene's expression value at next time point) is determined by $\sum_{i=1}^{N}d_{i}\cdot x_{i}[t]+k$ (the production effect of the *N *TFs at present time point and RNA polymerase II) and -*λ*·*y*[*t*] (the degradation effect of the target gene at present time point).

It has been shown that TF binding usually affects gene expression in a nonlinear fashion: below some level it has no effect, while above a certain level the effect may become saturated. This type of binding behavior can be modeled using a sigmoid function [10,39-42]. Therefore, we define *x*_*i*_[*t*] (the regulatory profile of TF*i *at time point *t*) as a sigmoid function of *z*_*i*_[*t*] (the gene expression profile of TF*i *at time point *t*):

(2) $$x_{i}[t]=f(z_{i}[t])=\frac{1}{1+\exp\left[-r\left(z_{i}[t]-A_{i}\right)\right]}$$

where *r *denotes the transition rate of the sigmoid function and *A*_*i *_denotes the mean of the gene expression profile of TF*i*.

### Estimating the parameters of the dynamic system model

We rewrite Equation (1) into the following regression form:

(3) $$\begin{array}{l} y[t+1]\equiv \phi [t]\cdot \theta +\varepsilon [t]\\ =\left[\begin{array}{ccccc} x_{1}[t] & \cdots & x_{N}[t] & 1 & -y[t] \end{array}\right]\cdot \left[\begin{array}{c} d_{1}\\ \vdots \\ d_{N}\\ k\\ \lambda \end{array}\right]+\varepsilon [t] \end{array}$$

where *φ*[*t*] = [*x*_1_[*t*] ⋯ *x*_*N*_[*t*] 1 - *y*[*t*]] denotes the regression vector and *θ *= [*d*_1 _⋯ *d*_*N *_*k λ*]^*T *^is the parameter vector.

From the gene expression data under heat shock in Caustion *et al*.'s study [18], it is easy to get the values of {*x*_*i*_[*t*_*v*_], *y*[*t*_*v*_]} for *i *∈ {1,2, ⋯, *N*}, *v *∈ {1, 2, ⋯, *M*}, where *M *is the number of the time points of a target gene's expression profile. Equation (3) at different time points can be put together as follows:

(4) $$\left[\begin{array}{c} y[t_{2}]\\ y[t_{3}]\\ \vdots \\ y[t_{M}] \end{array}\right]=\left[\begin{array}{c} \phi [t_{1}]\\ \phi [t_{2}]\\ \vdots \\ \phi [t_{M-1}] \end{array}\right]\cdot \theta +\left[\begin{array}{c} \varepsilon [t_{1}]\\ \varepsilon [t_{2}]\\ \vdots \\ \varepsilon [t_{M-1}] \end{array}\right]$$

For simplicity, we can further define the notations *Y *Φ and *e *to represent Equation (4) as follows:

(5) *Y *= Φ·*θ *+ *e*

The parameter vector *θ *can be estimated by the maximum likelihood (ML) method as follows [38]:

(6) $$\begin{array}{l} \hat{\theta }={\left(\Phi^{T}\Phi \right)}^{-1}\Phi^{T}Y\\ ={\left[\begin{array}{ccccc} {\hat{d}}_{1} & \cdots & {\hat{d}}_{N} & \hat{k} & \hat{\lambda } \end{array}\right]}^{T} \end{array}$$

Since *d*_*i *_stands for the regulatory ability of TF*i*, a small absolute value of *d*_*i *_means that TF*i *only has a small effect on the target gene's expression, while a large absolute value means that TF*i *has a large regulatory effect on the target gene's expression. We regard TF*i *to be a true regulator of the target gene if its regulatory ability *d*_*i *_is statistically significantly different from zero (i.e. |*d*_*i*_| ≫ 0). The test statistic $t=\frac{{\hat{d}}_{i}}{s\sqrt{u_{ii}}}$ a *t*-distribution with degree of freedom (*M *- 1) - (*N *+ 2) is used to assign a *p*-value for rejecting the null hypothesis *H*_*0*_: *d*_*i *_= 0, where *u*_*ii *_is the *i*th diagonal element of the matrix (Φ^*T*^Φ)^-1 ^and $s=\sqrt{\frac{{\left(Y-\Phi \cdot \hat{\theta }\right)}^{T}(Y-\Phi \cdot \hat{\theta })}{\left(M-1)-(N+2\right)}}$ is an unbiased estimator of *σ *(the standard deviation of the stochastic noise *ε*[*t*]) [24]. The *p*-value computed by the *i*-distribution is then adjusted by the Bonferroni correction to represent the true α level in the multiple hypothesis testing [24]. Then, TF*i *is said to be a true regulator of the target gene if the adjusted *p*-value *p*_*adjusted *_≤ 0.01.

## Authors' contributions

WSW developed the algorithm, performed the simulation and wrote the manuscript. WHL conceived the research topic, provided essential guidance and revised the manuscript. All authors read and approved the final manuscript.

## Supplementary Material

Additional file 1 Additional file 1 contains the detailed information of the 29 identified heat-inducible GRMs, the 182 identified heat-inducible genes, the 68 identified uncharacterized genes and the performance comparison of five module inference tools. Click here for file
